# Supplementary material for: Resolving the full spectrum of human genome variation using Linked-Reads
Source: Genome Res. 2019 Apr;29(4):635–45. doi: 10.1101/gr.234443.118 (PMC6442396; doi:10.1101/gr.234443.118)
Supplement: Supplemental Material [file supp_gr.234443.118_Supplemental_longranger-2.2.zip › longranger-2.2.tar.bz2!/lr2.2-public/lib/stan/cmdstan/stan/doc/api/html/namespacestan_1_1memory.html]

Stan: stan::memory Namespace Reference


|  |  |
| --- | --- |
|  | Stan  2.5.0  probability, sampling & optimization |


- Main Page
- Related Pages
- Namespaces
- Classes
- Files

- Namespace List
- Namespace Members

All Classes Namespaces Files Functions Variables Typedefs Enumerations Enumerator Friends Macros Pages

- stan
- memory

Classes |
Functions

stan::memory Namespace Reference

(Expert) Allocate, free, and manage underlying system memory.
More...

|  |  |
| --- | --- |
| Classes | |
| class | stack\_alloc |
|  | An instance of this class provides a memory pool through which blocks of raw memory may be allocated and then collected simultaneously. More... |
|  | |

|  |  |
| --- | --- |
| Functions | |
| template<typename T > | |
| bool | is\_aligned (T \*ptr, unsigned int bytes\_aligned) |
|  | Return `true` if the specified pointer is aligned on the number of bytes. More... |
|  | |

## Detailed Description

(Expert) Allocate, free, and manage underlying system memory.

## Function Documentation

template<typename T >

|  |  |  |  |
| --- | --- | --- | --- |
| bool stan::memory::is\_aligned | ( | T \* | *ptr*, |
|  |  | unsigned int | *bytes\_aligned* |
|  | ) |  |  |

Return `true` if the specified pointer is aligned on the number of bytes.

This doesn't really make sense other than for powers of 2.

Parameters
:   |  |  |
    | --- | --- |
    | ptr | Pointer to test. |
    | bytes\_aligned | Number of bytes of alignment required. |

Returns
:   `true` if pointer is aligned.

Template Parameters
:   |  |  |
    | --- | --- |
    | Type | of object to which pointer points. |

Definition at line 32 of file stack\_alloc.hpp.


---

[ Stan Home Page ]

*© 2011–2014,
Stan Development Team.*
